# LDL‐cholesterol and PCSK9 in patients with familial hypercholesterolemia: influence of PCSK9 variants under lipid‐lowering therapy

**DOI:** 10.1002/jcla.24056

**Published:** 2021-10-15

**Authors:** Masato Hamasaki, Naoki Sakane, Kazuo Hara, Kazuhiko Kotani

**Affiliations:** ^1^ Division of Community and Family Medicine Jichi Medical University Shimotsuke‐City Japan; ^2^ Division of Preventive Medicine Clinical Research Institute National Hospital Organization Kyoto Medical Center Kyoto‐City Japan; ^3^ Division of Endocrinology and Metabolism Jichi Medical University Saitama Medical Center Omiya‐City Japan

**Keywords:** gain‐of‐function mutation, genetic hypercholesterolemia, lipid‐lowering therapy, low‐density lipoprotein receptor, PCSK9 inhibitor, statins

## Abstract

**Background:**

Familial hypercholesterolemia (FH), an autosomal dominant genetic disease with the elevated levels of low‐density lipoprotein (LDL) cholesterol (LDL‐C), increases the risk of coronary artery disease (CAD). The proprotein convertase subtilisin/kexin type 9 (PCSK9) gene is associated with FH. There is a positive relationship between circulating LDL‐C and PCSK9 levels, a potential CAD condition, without lipid‐lowering therapy (LLT); however, we do not know whether their correlation exists in FH patients under LLT.

**Methods:**

This study compared the correlation of PCSK9 variants among patients with FH under LLT (n = 70; mean age, 53 years; male, 63%). LDLR, PCSK9 and APOB variants were analyzed using next‐generation sequencing.

**Results:**

The LDL‐C and PCSK9 levels in patients with gain‐of‐function (GOF) variants of PCSK9 (n = 7) were mostly similar to those in patients with LDLR variants (n = 17) or variant‐negative patients (n = 46). A significant positive correlation was observed between LDL‐C and PCSK9 levels in patients with GOF variants of PCSK9 (r = 0.79, *p* = 0.04), but not in patients with LDLR variants or variant‐negative patients.

**Conclusion:**

The LDL‐C‐PCSK9 correlation is suggested to be retained in FH patients with GOF variants of PCSK9 even under LLT, and these variants can be used as molecular markers for additional treatment with statins in FH patients.

## Graphical abstract

A significant positive correlation was observed between LDL‐C and PCSK9 levels in patients with gain‐of‐function variants of *PCSK9*, but not in patients with the other variants.

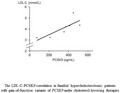

## 1 INTRODUCTION

Familial hypercholesterolemia (FH) is an autosomal dominant genetic disease that exhibits high levels of circulating low‐density lipoprotein (LDL) cholesterol (LDL‐C) and an increased risk of coronary artery disease (CAD).^1^ The most frequent causative gene, LDLR, encodes an LDL receptor (LDLR).^2^ In addition, APOB is a causative gene that encodes for an apolipoprotein B component of LDL, which is a ligand of LDLR, but the frequency of variants is very low in Asian countries.^3^, ^4^ Furthermore, proprotein convertase subtilisin/kexin type 9 (PCSK9) is another major causative gene of FH, encoding the recently discovered PCSK9, although the frequency of PCSK9 variants is reported to be comparatively low.^3^, ^4^, ^5^ PCSK9 promotes clathrin‐mediated endocytosis of LDLR by forming a complex with LDLR,^6^, ^7^ and LDLR degradation leads to high circulating LDL‐C levels. High circulating PCSK9 levels are a risk factor for CAD.^8^ Gain‐of‐function (GOF) variants of PCSK9 show high LDLR degradation activity, which leads to an additional increase in LDL‐C levels.^3^, ^9^, ^10^, ^11^, ^12^, ^13^ Thus, the risk of CAD is further increased by both PCSK9 levels and its variant‐related functions.

3‐Hydroxy‐3‐methylglutaryl coenzyme A (HMG‐CoA) reductase inhibitors, statins, are used as representative oral drugs in lipid‐lowering therapy (LLT). In general, statins reduce circulating LDL‐C levels by 30%–50%.^14^ During LLT, sterol regulatory element binding protein^2^ (SREBP2) responds to the reduction in cholesterol levels in hepatocytes, which upregulates LDLR expression, and the upregulated LDLR expression reduces circulating LDL‐C levels.^15^, ^16^ However, statins do not always reduce circulating LDL‐C levels to the targeted levels to prevent CAD in some FH patients.^14^ The insufficient reduction of LDL‐C by LLT may be influenced by the GOF variants of PCSK9 because SREBP2 also upregulates PCSK9 expression.^15^, ^16^ In this case, additional treatments, such as PCSK9 inhibitors, can be considered to further reduce LDL‐C levels.^17^, ^18^ Determining the GOF variants of PCSK9 may be useful not only for diagnosing FH, but also for assessing the response to drugs.

A positive correlation between LDL‐C and PCSK9 levels was observed “without LLT” in not only the general population but also in FH patients,^3^, ^19^, ^20^, ^21^ whereas this positive correlation disappeared in patients with hypercholesterolemia (in whom some FH patients might be included) “under LLT”.^20^, ^21^ However, when focusing only on FH patients, their correlation has not been well defined “under LLT” in relation to PCSK9 variants. Accordingly, the present study compared the correlation between circulating LDL‐C and PCSK9 levels by PCSK9 variants among FH patients under LLT.

## 2 MATERIALS AND METHODS

This cross‐sectional study enrolled 70 Japanese patients (mean age, 53 years; male, 63%) with FH under LLT. The patients were basically treated with statins at the maximum dose (i.e., 40 mg/day of atorvastatin, 4 mg/day of pitavastatin, 20 mg/day of rosuvastatin) depending on the situation of the respective patients. The patients who received statins plus Niemann–Pick C1‐like 1 inhibitor (10 mg/day of ezetimibe) or colestimide (4 mg/day) at the maximum dose were included. The definition of FH was based on the clinical diagnosis based on the FH criteria.^14^, ^22^ Patients with hepatic and renal dysfunction were excluded from the study. The study was approved by the Ethics Review Committee of Jichi Medical University (No. 20–002 and 20–022). The study was conducted in accordance with the Declaration of Helsinki, and written informed consent was obtained from all patients.

Serum total cholesterol and high‐density lipoprotein (HDL) cholesterol (HDL‐C) levels were, respectively, measured using enzymatic methods (intra‐assay coefficient of variation [CV] and inter‐assay CV; 0.4% and 1.1% in total cholesterol, 1.7% and 2.4% in HDL‐C). Serum triglycerides levels were measured with total glycerol using enzymatic methods (intra‐assay CV and inter‐assay CV; 0.5% and 0.4%).^23^, ^24^ Serum LDL‐C levels were calculated using the Friedewald equation (in the present study, the triglycerides values of all patients were under 4.5 mmol/L).^25^ Serum PCSK9 levels were measured using a PCSK9 Quantikine ELISA Kit (R&D Systems, Minneapolis, MN, USA).

LDLR, PCSK9 and APOB variants were examined using next‐generation sequencing using 50 ng of genomic DNA via NextSeq500 (Illumina, San Diego, CA, USA). Library preparation was performed using a TruSight One sequencing panel (Illumina, San Diego, CA, USA). Variant data were obtained using the ANNOVAR tool,^26^ and the analyzed variants of amino acid substitutions or splice regions were interpreted using the ClinVar database.^27^

One‐way ANOVA and Fisher's exact test were used to analyze the differences in the measured variables among the groups. The Pearson correlation test was used to analyze the correlation between LDL‐C and PCSK9 levels in each group. Comparison tests using Fisher's z‐transformation were performed for the correlation coefficients between the groups with the LDLR variant, the GOF variants of PCSK9 and variant‐negative patients. The triglycerides values were log‐transformed in the analyses because of their skewed distributions. Statistical significance was set at *p* < 0.05. All statistical analyses were performed using the R statistical package, version 3.3.0. (https://www.R‐project.org/).

## 3 RESULTS

The characteristics of the study patients are summarized in Table 1. Two heterozygous GOF variants of PCSK9, p.V4I (n = 1) and p.E32K (n = 6), were observed in seven patients (mean age, 55 years; male, n = 1). These two variants are reported to be common variants and have been reported to induce high LDL‐C levels ^3^, ^11^, ^28^, ^29^. Heterozygous variants of LDLR were observed in 17 patients (mean age, 43 years; male, n = 13). Variants of APOB were not observed in the present study, and patients without PCSK9 or LDLR variants, a variant‐negative group, were observed (mean age, 56 years; male, n = 30).

**TABLE 1:** Characteristics of the studied patients by gene variants

Variables	*LDLR+* (n = 17)	*PCSK9+* (n = 7)	Variant‐negative (n = 46)	*P*‐value
T‐Chol, mmol/L	6.60 ± 1.51	6.14 ± 0.93	5.94 ± 1.17	0.17
Triglycerides, mmol/L	1.15 (0.86–1.51)	2.15 (2.04–2.24)	1.30 (1.01–1.68)	0.08
HDL‐C, mmol/L	1.50 ± 0.31	1.33 ± 0.34	1.74 ± 0.53	0.05
LDL‐C, mmol/L	4.13 ± 1.16	4.06 ± 0.78	3.55 ± 0.97	0.10
PCSK9, ng/mL	385 ± 126	342 ± 150	330 ± 84	0.17
Lipid‐lowering therapy				0.08
Statins, n (%)	15 (88%)	6 (86%)	45 (98%)	‐
Colestimide, n (%)	0 (0%)	1 (14%)	0 (0%)	‐
Statins +ezetimibe, n (%)	2 (12%)	0 (0%)	1 (2%)	‐

Note

Data are shown as the mean ±standard deviation or median (interquartile range). The triglycerides values are displayed as median (interquartile range) because of the skewed distribution. *P*‐values analyzed by one‐way ANOVA (each lipid variable and PCSK9) and by Fisher's exact test (lipid‐lowering therapy).

Abbreviations: HDL‐C, high‐density lipoprotein cholesterol; LDL‐C, low‐density lipoprotein cholesterol; LDLR, low‐density lipoprotein receptor; *LDLR*+, patients with *LDLR* variants; PCSK9, proprotein circulating convertase subtilisin/kexin type 9; *PCSK9*+, patients with *PCSK9* variants; T‐Chol, total cholesterol.

As shown in Table 1, the LDL‐C and PCSK9 levels were similar among the groups. The HDL‐C levels tended to be low and the triglycerides levels tended to be high in patients with PCSK9 variants, but the levels were not significantly different among the groups. The two variants, p.V4I and p.E32K, showed similar LDL‐C (mean, 4.28 and 4.01 mmol/L, respectively) and PCSK9 levels (mean, 411 and 330 ng/mL, respectively). The prevalence of drugs used in LLT were not significantly different among the groups.

**FIGURE 1:**
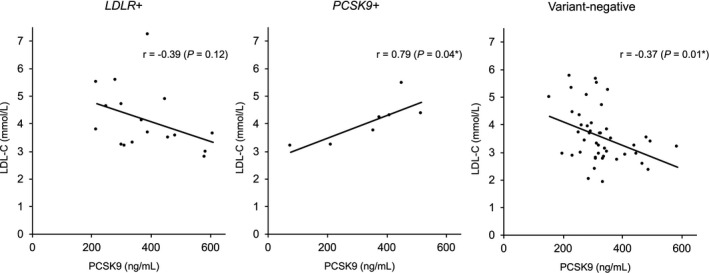
Correlations between LDL‐C and PCSK9 levels by gene variants. LDL‐C, low‐density lipoprotein cholesterol; LDLR, low‐density lipoprotein receptor; PCSK9, proprotein circulating convertase subtilisin/kexin type 9; FH, familial hypercholesterolemia; *LDLR*+, patients with *LDLR* variants; *PCSK9*+, patients with *PCSK9* variants; r values and *P*‐values analyzed by Pearson correlation test; ^*^ significance (correlation coefficient), <0.05

As shown in Figure 1, LDL‐C and PCSK9 levels in patients with PCSK9 variants were significantly positively correlated (r = 0.79, *p* = 0.04). In contrast, LDL‐C and PCSK9 levels were significantly negatively correlated in variant‐negative patients (r = −0.37, *p* = 0.01) and were insignificantly but negatively correlated in patients with LDLR variants (r = −0.39, *p* = 0.12). In addition, the correlation coefficient was significantly different between patients with LDLR variants and GOF variants of PCSK9 (*p* = 0.01) as well as between patients with GOF variants of PCSK9 and variant‐negative patients (*p* < 0.01).

## 4 DISCUSSION

In the present study, there was a significant positive correlation between LDL‐C and PCSK9 levels in FH patients with GOF variants of PCSK9 under LLT. In contrast, their positive correlation was not observed in the patients with LDLR variants or in variant‐negative patients. This is the first study to demonstrate that the correlation between LDL‐C and PCSK9 levels could be modulated by GOF variants of PCSK9 among FH patients “under LLT.” This implies that the GOF variants of PCSK9 may be considered as a molecular marker in additional treatments in FH patients.

The mild negative correlation was observed between LDL‐C and PCSK9 levels in the patients without GOF variants of PCSK9 under LLT. Although their mild correlation was similarly observed in previous studies on patients with hypercholesterolemia under LLT, PCSK9 variants were not examined in these studies.^20^, ^21^ Some possible explanations for their mild correlations are considered. For instance, LLT, such as statin therapy, reduces circulating LDL‐C levels and increases circulating PCSK9 levels in the opposite direction^15^, ^16^, ^30^; namely, LLT weakens the positive correlation observed in individuals “without LLT.” The mild correlation is also thought to be owing to different responses to LLT among individuals.^20^, ^21^ Furthermore, adopting various lifestyle modifications, including diet and exercise, as a treatment additional to LLT for hypercholesterolemia, may partly weaken the correlation in patients under LLT.^31^, ^32^, ^33^

The notable finding of the present study is that the positive correlation between LDL‐C and PCSK9 levels in FH patients with GOF variants of PCSK9 was retained even under LLT, similar to their positive correlation in FH patients “without LLT”.^19^, ^34^ PCSK9 variants, observed in the present study, were the GOF type,^3^, ^11^, ^28^ which promotes the rapid turnover of LDLR (the turnover accelerates the degradation of LDLR by its high affinity with LDLR) compared to non‐PCSK9 variants,^12^, ^35^, ^36^ resulting in a failure of LDL uptake in hepatocytes. This process leads to high levels of circulating LDL‐C. This process can be observed even under LLT. Namely, in the present study setting, the positive correlation between LDL‐C and PCSK9 levels under LLT might only appear in FH patients with GOF variants of PCSK9.

A positive correlation between LDL‐C and PCSK9 levels is a condition of CAD risk,^3^, ^34^ and again, in the present study, such a correlation was seen in FH patients with GOF variants of PCSK9 even under LLT. The inhibition of PCSK9 is reasonable for the reduction of circulating PCSK9 levels, as elevated by statins.^15^, ^16^ Thus, the addition of PCSK9 inhibitors to statins is assumed to be a suitable treatment to remove this positive correlation, especially in the GOF variants of PCSK9.

The present study had some limitations. First, the sample size was relatively small. Second, the cross‐sectional study might not have fully determined the causality. Third, although lifestyle factors are known to modify LDL‐C and PCSK9 levels,^31^, ^32^, ^33^ these factors were not examined in this study. Further detailed analyses are needed to corroborate the results of the present study.

## 5 CONCLUSION

In the present study, circulating LDL‐C and PCSK9 levels were positively correlated in FH patients with GOF of PCSK9 variants under LLT. The LDL‐C‐PCSK9 correlation is a potential CAD condition, and the variant detection may be useful for additional treatments on statins, such as PCSK9 inhibitors, in FH patients.

## CONFLICT OF INTERESTS

M.H. also works at Eiken Chemical Co., Ltd.

## AUTHOR CONTRIBUTIONS

M.H. and K.K. were involved in conceptualization and formal analysis; N.S. and K.H. participated in validation. M.H. and N.S. carried out investigation; M.H. wrote and prepared the original draft; K.H. and K.K. had contributed to writing, reviewing and editing, and supervision. All authors have read and agreed to the published version of the manuscript.

## Data Availability

The data of this study are available from the corresponding author upon reasonable request.
